# COVID-19 associated hospitalization in 571 patients with fibromyalgia—A population-based study

**DOI:** 10.1371/journal.pone.0261772

**Published:** 2021-12-30

**Authors:** Mor Amital, Niv Ben-Shabat, Howard Amital, Dan Buskila, Arnon D. Cohen, Daniela Amital

**Affiliations:** 1 The Adelson School of Medicine, Ariel University, Ariel, Israel; 2 Department of Medicine ’B’ & Zabludowicz Center for Autoimmune Diseases, Sheba Medical Center, Tel-Hashomer, Ramat-Gan, Israel; 3 Sackler Faculty of Medicine, Tel-Aviv University, Tel-Aviv, Israel; 4 Faculty of Health Sciences, Siaal Research Center for Family Medicine and Primary Care, Beer Sheva, Israel; 5 Ben Gurion University of the Negev, Beer Sheva, Israel; 6 Department of Quality Measurements and Research, Clalit Health Services, Tel-Aviv, Israel; 7 Division of Psychiatry, Barzilai Medical Center, Ashkelon, Israel; Capital Medical University, CHINA

## Abstract

**Objective:**

To identify predicators of patients with fibromyalgia (FM) that are associated with a severe COVID-19 disease course.

**Methods:**

We utilized the data base of the Clalit Health Services (CHS); the largest public organization in Israel, and extracted data concerning patients with FM. We matched two subjects without FM to each subject with FM by sex and age and geographic location. Baseline characteristics were evaluated by t-test for continuous variables and chi-square for categorical variables. Predictors of COVID-19 associated hospitalization were identified using univariable logistic regression model, significant variables were selected and analyzed by a multivariable logistic regression model.

**Results:**

The initial cohort comprised 18,598 patients with FM and 36,985 matched controls. The mean age was 57.5± 14.5(SD), with a female dominance of 91%. Out of this cohort we extracted the study population, which included all patients contracted with COVID-19, and consisted of 571 patients with FM and 1008 controls. By multivariable analysis, the following variables were found to predict COVID-19 associated hospitalization in patients with FM: older age (OR, 1.25; CI, 1.13–1.39; p<0.001), male sex (OR, 2.63; CI, 1.18–5.88; p<0.05) and hypertension (OR, 1.75; CI, 1.04–2.95; p<0.05).

**Conclusion:**

The current population-based study revealed that FM *per se* was not directly associated with COVID-19 hospitalization or related mortality. Yet classical risk factors endangering the general population were also relevant among patients with FM.

## Introduction

Fibromyalgia (FM) is characterized by widespread pain, unrefreshing sleep, fatigue, and cognitive impairment. Headaches, sensitive bladder, irritable bowel disease and temporal-mandibular discomfort, are also often encountered in these patients [1].

FM is the second most common rheumatological condition with a female dominance ranging between 4–9:1 [2].

It is accepted that FM may be triggered by various infectious diseases [3, 4]; Buskila et al [5] revealed high rates of FM (16%) in hepatitis C virus (HCV) patients, compared to subjects with non-HCV-related cirrhosis (3%), similarly, hepatitis B carriage appears to increase the risk of FM [6]. Accumulating evidence suggest that an imbalance between pro-inflammatory and anti-inflammatory cytokines may facilitate the emergence of neuropathic pain [7]. Furthermore, elevated serum concentrations of IL-6, IL-8 and IL-1RA were detected in patients with FM [7–9]. These proinflammatory cytokines up regulate the expression of pain mediators such as substance P, which decrease pain threshold.

The COVID19 global pandemic created numerous global challenges among them in particular is the overwhelming hospitalization rates of patients with the disease that saturated the capacities of our health systems. After an extended stay in these designated medical units, patients recovering from severe COVID-19 report a long and painful convalescence [10]. Since the management of COVID-19 patients is primarily supportive, many patients experience a distressing course, which is sometimes on the verge of death. Such experiences often results in consequent post-traumatic ideation, high levels of anxiety and consequent development of mood disorders [11]

The aim of our study was to analyze the patterns of morbidity and mortality in a large cohort of patients with FM who were positive with the SARS COV-2 and to assess whether they were more vulnerable to its deleterious outcomes. In order to do so we used the Clalit Health Care Services (CHS) database the largest Health Maintenance Organization (HMO) in Israel.

## Methods

### Study design and study population

This is a retrospective longitudinal study, investigating predictors for COVID-19 associated hospitalization in patients with FM. The current study was approved by the institutional review board (IRB) of the CHS. CHS is the largest HMO in Israel with widespread primary, secondary, and tertiary medical services covering almost 5 million citizens. CHS database enables access to a wide range of clinical data, as it receives information from various live logistical, pharmaceutical and medical sources. The current primary cohort includes all patients with FM who were registered between the years 2002–2018 (n = 18,598). We also formed a control group, electing for each FM patient two controls adjusted by age, gender and geographic location (n = 36,985). The controls were selected by a computerized algorithm that was applied on the database. The diagnosis of FM did not appear in their computerized medical records. The algorithm was provided selection of individuals that will have a similar distribution of their demographic parameters. Based on this primary cohort we conducted a subgroup analysis including all patients who were diagnosed with COVID-19 since the beginning of the pandemic in Israel until 21 of October 2020. A total of 571 patients with FM and 1008 controls were included in this analysis.

### COVID-19-related variables

COVID-19 diagnosis was based on confirmation of all cases by US Food and Drug Administration (FDA) approved molecular tests. COVID-19-associated hospitalization was defined as patients admitted to intensive care units, internal medicine, or pulmonary inpatients wards. The severity of non-hospitalized COVID-19 patients, who did not require admission to a medical facility was termed for the purpose of this study as “subclinical”.

### Definition of FM-related and comorbidity variables

Patients were defined as having FM if their medical records contained at least one diagnosis by a physician. Chronic comorbidities were obtained by the chronic registry of CHS. All comorbidities were encoded prior to the onset of the pandemic. Structural heart disease was defined as either valvular heart disease or cardiomyopathy. Atherosclerotic related disease was defined as one of the following: ischemic heart disease, peripheral vascular disease, cerebrovascular accident (CVA).

### Statistical analysis

Baseline characteristics were described by means and standard deviations (SD) for continuous variables, categorical variables were characterized by percentages. Differences between groups were calculated by t-test or Mann-Whitney test for continues variables and chi-square for categorical variables. Comorbidities were registered prior to the onset of the pandemic. The association between comorbidities variables and COVID-19 associated hospitalization were evaluated using univariable logistic regression and were reported as odd ration (OR) with 95% confidence intervals (CIs). Significant variables (p<0.05) were included in the multivariable logistic regression and were reported as odd ration (OR) with 95% confidence intervals (CIs).

## Results

### Characteristics of the study population

The initial cohort comprised 18,598 patients with FM and 36,985 controls matched by age, gender, and geographical location. The mean age was 57.5± 14.5(SD), with a female dominance of 91%. The secondary subgroup analysis cohort consisted of 571 patients with FM and 1008 controls with a diagnosis of COVID-19. The mean age of the FM group was 56.2± 13.7 (SD) and 92.6% were females. The mean age of the control group was 54.8± 13.3(SD) with 90.6% females. The two groups did not differ significantly by sex, ethnicity, and socioeconomic status. However, a significantly difference was measured by age (FM-56.2 vs. non-FM-54.8 years, p<0.05) and BMI (FM 30.2 vs. non-FM 28.8, p<0.01). In addition, the FM COVID-19 group had higher rates of comorbidities such as diabetes, hyperlipidemia, asthma, atherosclerosis related diseases, rheumatoid arthritis, SLE, depression and anxiety (Table 1).

**Table 1:** Basic characteristics of study population.

	Initial cohort			COVID-19 positive cohort		
	Fibromyalgia (n = 18,598)	Controls (n = 36,985)	p-value	Fibromyalgia (n = 571)	Controls (n = 1008)	p-value
Age, mean (SD)	57.5 (14.1)	57.5 (14.1)	0.758	56.2 (13.7)	54.8 (13.3)	0.044
BMI, mean (SD)	29.1 (6.3)	28.1 (6.1)	<0.001	30.2 (6.3)	28.8 (6.1)	<0.001
Females, n(%)	16,920 (91%)	33,651 (91%)	0.97	529 (92.6%)	913 (90.6%)	0.164
Ethnicity, (n%)			0.99			0.222
Jewish	14,811 (79.6%)	29,451 (79.6%)		395 (69.2%)	719 (71.3%)	
Arab	3,473 (18.7%)	6,915 (18.7%)		155 (27.1%)	240 (23.8%)	
Ultraorthodox Jews	314 (1.7%)	619 (1.7%)		21 (3.7%)	49 (4.9%)	
Socioeconomic status, n(%)			0.97			0.471
Low	8,414 (45.3%)	16,770 (45.4%)		318 (55.9%)	540 (53.8%)	
Intermediate	7,168 (38.6%)	14,235 (38.6%)		183 (32.2%)	353 (35.2%)	
High	2,985 (16.1%)	5,918 (16.0%)		68 (4.3%)	111 (11.1%)	
Hypertension, n(%)	12,419 (33.2%)	26,238 (29.1%)	<0.001	181 (31.7%)	276 (27.4%)	0.073
Diabetes, n(%)	4,183 (22.5%)	7,261 (19.6%)	<0.001	144 (25.2%)	197 (19.5%)	<0.01
Hyperlipidemia, n(%)	11,192 (60.2%)	19,176 (51.8%)	<0.001	327 (57.3%)	492 (48.8%)	<0.001
Smoking (ever), n(%)	6,913 (37.2%)	11,106 (30%)	<0.001	160 (28%)	214 (21.2%)	<0.01
Asthma, n(%)	2,244 (12.1%)	2,424 (6.1%)	<0.001	64 (11.2%)	67 (6.6%)	<0.01
COPD, n(%)	982 (5.3%)	946 (2.6%)	<0.001	18 (3.2%)	18 (1.8%)	0.113
h/o tuberculosis, n(%)	24 (0.1%)	31 (0.1%)	0.11	0	3 (0.3%)	0.557
^a^Atherosclerosis-related disease, n(%)	2,293 (12.3%)	3,008 (8.1%)	<0.001	71 (12.4%)	73 (7.2%)	<0.001
^b^Structural heart disease, n(%)	1,262 (6.8%)	1,632 (4.4%)	<0.001	34 (6%)	40 (4%)	0.083
Chronic renal failure, n(%)	482 (2.6%)	859 (2.3%)	0.053	9 (1.6%)	16 (1.6%)	1.000
Cirrhosis, n(%)	75 (0.4%)	87 (0.2%)	<0.001	2 (0.4%)	2 (0.2%)	0.623
Malignancy, n(%)	2,187 (11.8%)	3,593 (9.7%)	<0.001	54 (9.5%)	69 (6.8%)	0.064
Rheumatoid arthritis, n(%)	1,039 (5.6%)	492 (1.3%)	<0.001	42 (7.4%)	15 (26.3%)	<0.001
SLE, n(%)	172 (0.9%)	110 (0.3%)	<0.001	9 (1.6%)	5 (0.5%)	<0.05
Crohn’s, n(%)	154 (0.8%)	157 (0.4%)	<0.001	3 (0.5%)	1 (0.1%)	0.138
Ulcerative colitis, n(%)	118 (0.6%)	133 (0.4%)	<0.001	2 (0.4%)	2 (0.2%)	0.623
Celiac, n(%)	138 (0.7%)	137 (0.4%)	<0.001	2 (0.4%)	5 (0.5%)	0.675
Cushing disease, n(%)	39 (0.2%)	24 (0.1%)	<0.001	2 (0.4%)	1 (0.1%)	0.298
Depression, n(%)	5,248 (28.2%)	3,531 (9.5%)	<0.001	140 (24.5%)	77 (7.6%)	<0.001
Anxiety, n(%)	3,873 (20.8%)	2,814 (7.6%)	<0.001	110 (19.3%)	58 (5.8%)	<0.001
COVID-19 positive, n(%)	571 (3.1%)	1,008 (2.8%)	0.026	-	-	
COVID-19 duration, median (IQR)	-	-	-	18.0 (12–31)	17.0 (12–31)	0.600
COVID-19 hospitalization, n(%)	81 (0.4%)	125 (0.3%)	0.075	81 (14.2%)	125 (12.4%)	0.313
Hospitalization duration, median (IQR)	-	-	-	4.0 (2–9)	3 (2–7)	0.122
COVID-19 death, n(%)	10 (0.1%)	17 (0.0%)	0.687	9 (1.6%)	13 (1.3%)	0.659
COVID-19 recovery, n(%)	411 (2.2%)	741(2.0%)	0.107	393 (68.8%)	710 (70.4%)	0.530

n-number, COPD-chronic obstructive pulmonary disease, SLE- Systemic Lupus Erythematosus, BMI-body mass index, IQR- interquartile range

^a^Atherosclerotic related disease was defined as one of the following: ischemic heart disease, peripheral vascular disease, cerebrovascular accident (CVA)

^b^ Structural heart disease was defined as either valvular heart disease or cardiomyopathy.

Regarding COVID-19 associated hospitalization and mortality, no significant differences were found between the FM and non-FM COVID-19 patients (p = 0.263, p = 0.659) (Table 1).

### Factors associated with COVID-19 hospitalization in fibromyalgia patients

Table 2 demonstrates factors associated with COVID-19 hospitalization. All variables that were significant in the univariate analysis, were included in the multivariable model.

**Table 2:** Factors associated with COVID-19 hospitalization in patients with fibromyalgia, obtained from a univariate and a multivariate logistic-regression analysis.

	Frequency	Univariate OR	95% CI	p-value	Multivariate OR	95% CI	p-value
Age (5-year increment)	63.4±12.9	1.28	1.16–1.41	<0.001	1.25	1.13–1.39	<0.001
Fibromyalgia^a^	81 (14.2%)	1.17	0.86–1.60	NS			
BMI (5-kg/m^2^-increment)	31.5±6.5	1.19	1.00–1.42	<0.05			
Male gender	10 (12.3%)	2.02	0.95–4.28	NS	2.63	1.18–5.88	<0.05
Arab ethnicity (vs Jewish ethnicity)	20 (24.7%)	0.84	0.49–1.45	NS			
Ultraorthodox ethnicity (vs Jewish ethnicity)	2 (2.5%)	1.05	0.44–2.55	NS			
Low SES (vs intermediate-high)	44 (54.3%)	0.93	0.58–1.49	NS			
Hypertension	41 (50.6%)	2.56	1.59–4.13	<0.001	1.75	1.04–2.95	<0.05
Diabetes	35 (43.2%)	2.66	1.63–4.33	<0.001			
Hyperlipidemia	62 (76.5%)	2.77	1.61–4.77	<0.001			
Asthma	10 (12.3%)	1.14	0.55–2.37	NS			
COPD	6 (7.4%)	3.19	1.16–8.75	<0.05			
^b^Atherosclerosis-related disease	19 (23.5%)	2.58	1.43–4.65	<0.01			
^c^Structural heart disease	8 (9.9%)	1.96	0.85–4.48	NS			
Chronic renal failure	4 (4.9%)	5.04	1.32–19.18	<0.05			
Cirrhosis	0	-	-				
Malignancy	12 (14.8%)	1.85	0.93–3.67	NS			
Rheumatoid arthritis	12 (14.8%)	2.67	1.30–5.45	<0.01			
SLE	1 (1.2%)	0.75	0.09–6.10	NS			
IBD	1 (1.2%)	2.03	0.21–19.75	NS			
Depression	25 (30.9%)	1.46	0.87–2.44	NS			
Anxiety	18 (22.2%)	1.24	0.67–2.19	NS			

Only variables demonstrating *P*<0.050 in the univariate analysis were subject to inclusion in the multivariate logistic regression model; Variables with cell sizes <5 by status were collapsed to ensure sufficient power in the adjusted model.

COPD-chronic obstructive pulmonary disease, SLE-Systemic Lupus Erythematosus, BMI-body mass index, IBD-Inflammatory bowel diseases (Ulcerative colitis, Crohn).

^a^ Model for this variable included entire study population.

^b^ Atherosclerotic related disease was defined as one of the following: ischemic heart disease, peripheral vascular disease, cerebrovascular accident (CVA)

^c^ Structural heart disease was defined as either valvular heart disease or cardiomyopathy.

In the multivariable analysis, the following variables were found to predict COVID-19 associated hospitalization: older age (OR, 1.25; CI, 1.13–1.39; p<0.001), male sex (OR, 2.63; CI, 1.18–5.88; p<0.05) and hypertension (OR, 1.75; CI, 1.04–2.95; p<0.05). All the other above-mentioned comorbidities did not contribute to a more severe COVID-19 disease in patients with FM.

### Factors associated with COVID-19 hospitalization in controls patients

Factors associated to COVID-19 hospitalization are represented in Table 3. All variables that were significant in the univariable model, were included in the multivariable model. In this analysis the following variables predicted COVID-19 associated hospitalization: older age (OR, 1.33; 95% CI; p<0.001), BMI (OR, 1.22; 95% CI; p<0.01), COPD (OR, 3.85; 95% CI; p<0.01) and chronic renal failure (OR, 6.45; 95% CI; p<0.001). A similar analysis for the combined cohort is presented in (S1 Table).

**Table 3:** Factors associated with COVID-19 hospitalization in controls, obtained from a univariate and a multivariate logistic-regression analysis.

	Frequency	Univariate OR	95% CI	p-value	Multivariate OR	95% CI	p-value
Age (5-year increment)	63.7 ± 13.3	1.36	1.26–1.47	<0.001	1.33	1.22–1.44	<0.001
BMI (5-kg/m^2^-increment	30.4± 7.5	1.26	1.09–1.46	<0.001	1.22	1.05–1.43	<0.01
Male gender	19 (15.2%)	1.90	1.11–3.27	<0.01			
Arab ethnicity (vs Jewish ethnicity)	35 (29.4%)	1.29	0.84–1.97	NS			
Ultraorthodox ethnicity (vs Jewish ethnicity)	6 (6.7%)	0.60	0.14–2.64	NS			
Low SES (vs intermediate-high)	44 (54.3%)	0.80	0.55–1.16	NS			
Hypertension	63 (50.4%)	3.20	2.18–4.69	<0.001			
Diabetes	43 (34.4%)	2.48	1.65–3.73	<0.001			
Hyperlipidemia	86 (68.8%)	2.59	1.73–3.87	<0.001			
Asthma	14 (11.2%	1.97	1.06–3.68	<0.05			
COPD	8 (6.4%)	5.97	2.31–15.43	<0.001	3.85	1.41–10.51	<0.01
^a^Atherosclerosis-related disease	15 (12%)	1.94	1.06–3.54	<0.05			
^b^Structural heart disease	10 (8%)	2.47	1.18–5.19	<0.05			
Chronic renal failure	10 (8%)	12.71	4.54–35.62**	<0.001	6.45	2.14–19.58	<0.001
Cirrhosis	1 (0.8%)	7.11	0.44–114	NS			
Malignancy	10 (8%)	1.21	0.60–2.44	NS			
Rheumatoid arthritis	2 (1.6%)	1.01	0.24–4.88	NS			
SLE	4 (3.2%)	29.16	3.23–263	<0.01			
IBD	2 (1.6%)	14.34	1.29–159				
Depression	17 (13.6%)	2.16	1.21–3.84				
Anxiety	11 (8.8%)	1.72	0.86–3.40				

Only variables demonstrating *P*<0.050 in the univariate analysis were subject to inclusion in the multivariate logistic regression model; Variables with cell sizes <5 by status were collapsed to ensure sufficient power in the adjusted model.

COPD-chronic obstructive pulmonary disease, SLE-Systemic Lupus Erythematosus, BMI-body mass index, IBD-Inflammatory bowel diseases (Ulcerative colitis, Crohn).

^a^ Atherosclerotic related disease was defined as one of the following: ischemic heart disease, peripheral vascular disease, cerebrovascular accident (CVA)

^b^ Structural heart disease was defined as either valvular heart disease or cardiomyopathy.

## Discussion

Many of the symptoms of the “post infectious” disorders resemble those of FM, such as fatigue, myalgia and sleep disturbances. Several studies shed light on the role of infectious diseases on the pathogenesis of FM [3–6].

The current population-based study revealed that FM *per se* was not directly associated with COVID-19 hospitalization or related mortality. In both groups age was statistically associated with COVID-19 related hospitalization, in addition to male sex and hypertension in the FM group and BMI, coexisting COPD and chronic renal failure in the non-FM group. Although the majority of the subjects were females, male sex was found to be associated to COVID-19 hospitalization in the FM group. Even though FM is more common in women, Buskila et al [12] described that men with FM had more severe symptoms compared to matched women. Regrading this subject, men with COVID-19 tend to develop more serious outcomes and higher rates of death compared to women [13].

As expected, FM patients had higher rates of depression (p<0.001) and anxiety (p<0.001). These findings are in concordance with previous studies suggesting that FM patients have higher rates of psychiatric conditions [14–21]. According to the Canadian Community Health Survey database, the prevalence of major depression was three times higher in subjects with FM than in those without the condition [22]. An Italian group observed a correlation between lifetime exposure to traumatic events and post-traumatic symptoms, to the severity of FM symptoms [15]. In this 52.8% of patients with FM met the criteria of major depression and 67.1% of panic disorder. In this study We found that depression was significantly associated with COVID-19 hospitalization in the non-FM group according to the univariable analysis model (OR, 2.16; 95% CI; p<0.01), whereas in the FM group it had no additional impact. This may be attributed to the fact that the FM group had significantly higher rates of depression compared to the control group at the baseline analysis (p<0.001). Thus, depression is not a discriminative feature as many of the FM patients have comorbid depression.

As could be expected the FM group in our study had higher proportions of rheumatoid arthritis and systemic lupus erythematosus. In several studies, high rates of FM diagnoses had been found in patients with rheumatic diseases [18, 19, 23]. Torrente-Segarra et al. [20] examined 3,591 patients with SLE in a cross sectional study and showed that the rates of FM diagnoses in SLE patients was significantly higher compared to the general population, especially in later stages of the disease. Wolfe and Michaud [21] reported that out of 11,866 patients with RA, 2078 (17.5%) fulfilled the criteria of FM diagnoses, this subgroup of patients had a more severe form of RA and higher major comorbid conditions.

In the current study FM patients had higher rates of diabetes and hyperlipidemia than the control group. Several studies reported higher proportions of FM in patients with diabetes, ranging from 9 to 23.3% [24, 25]. Based on a Taiwanese database of 47,270 patients with FM and 189,112 matched controls, the overall risk of stroke was 1.25-fold higher in FM group compared to the non-FM group [26]. Our study also found that FM patients had higher proportion of conventional cardiovascular risk factors such as diabetes, hyperlipidemia and hypertension.

One cannot exclude that co-linearity might have obscured the outcomes of this study. Our study corroborates that COPD, BMI, male gender, chronic renal failure, hypertension and age are established risk factors that contribute to a severe form of COVID-19 [27–30].

In conclusion, our study has shown that FM does not contribute as an independent risk factor to severe COVID-19 related morbidity or mortality. As infectious diseases may facilitate the appearance of FM, further research should be done on the effects of "post COVID-19 syndrome" and its relation to the emergence of FM.

## Supporting information

S1 Table Factors associated with COVID-19 hospitalization in the entire study population, obtained from a univariate and a multivariate logistic-regression analysis. (DOCX)
