# Supplementary material for: COVID-19 associated hospitalization in 571 patients with fibromyalgia—A population-based study
Source: PLoS One. 2021 Dec 30;16(12):e0261772. doi: 10.1371/journal.pone.0261772 (PMC8717981; doi:10.1371/journal.pone.0261772)
Supplement: S1 Table — (DOCX) [file pone.0261772.s001.docx]

***S1 Table***: Factors associated with COVID-19 hospitalization in the entire study population, obtained from a univariate and a multivariate logistic-regression analysis

|  | Univariate OR | 95% CI | p-value | Multivariate OR | 95% CI | p-value |
| --- | --- | --- | --- | --- | --- | --- |
| Age (5-year increment) | 1.33 | 1.25-1.41^**^ | <0.001 | 1.29 | 1.20-1.37 | <0.001 |
| Fibromyalgia | 1.17 | 0.86-1.60 | NS |  |  |  |
| BMI (5-kg/m^2^-increment) | 1.24 | 1.11-1.39 | <0.001 | 1.16 | 1.03-1.32 | <0.05 |
| Male gender | 1.92 | 1.24-2.98 | <0.01 | 1.87 | 1.15-3.03 | <0.05 |
| Arab ethnicity (vs Jewish ethnicity) | 1.01 | 0.79-1.53 | NS |  |  |  |
| Ultraorthodox ethnicity (vs Jewish ethnicity) | 0.88 | 0.41-1.87 | NS |  |  |  |
| Low SES (vs intermediate-high) | 0.27 | 0.63-1.14 | NS |  |  |  |
| Hypertension | 2.95 | 2.18-3.97 | <0.001 |  |  |  |
| Diabetes | 2.57 | 1.88-3.51 | <0.001 | 1.46 | 1.04-2.07 | <0.05 |
| Hyperlipidemia | 2.67 | 1.94-3.68 | <0.001 |  |  |  |
| Asthma | 1.56 | 0.98-2.49 | NS |  |  |  |
| COPD | 4.48 | 2.25-8.90 | <0.001 | 2.36 | 1.11-4.99 | <0.05 |
| ^a^Atherosclerosis-related disease | 2.27 | 1.50-3.44 | <0.001 |  |  |  |
| ^b^Structural heart disease | 2.25 | 1.30-3.91 | <0.01 |  |  |  |
| Chronic renal failure | 9.03 | 4.04-20.17 | <0.001 | 3.58 | 1.52-8.48 | <0.01 |
| Cirrhosis | 2.23 | 0.23-21.52 | NS |  |  |  |
| Malignancy | 1.51 | 0.93-2.45 | NS |  |  |  |
| Rheumatoid arthritis | 2.25 | 1.21-4.20 | <0.05 |  |  |  |
| SLE | 3.77 | 1.25-11.36 | <0.05 | 5.34 | 1.60-17.77 | <0.01 |
| IBD | 5.06 | 1.12-22.76 | <0.05 | 8.31 | 1.50-46.11 | <0.05 |
| Depression | 1.75 | 1.21-2.55 | <0.01 |  |  |  |
| Anxiety | 1.45 | 0.95-2.24 | NS |  |  |  |

Only variables demonstrating *P*<0.050 in the univariate analysis were subject to inclusion in the multivariate logistic regression model; Variables with cell sizes <5 by status were collapsed to ensure sufficient power in the adjusted model.

COPD-chronic obstructive pulmonary disease, SLE-Systemic Lupus Erythematosus, BMI-body mass index, IBD-Inflammatory bowel diseases (Ulcerative colitis, Crohn), NS non-significant.

^a^ Atherosclerotic related disease was defined as one of the following: ischemic heart disease, peripheral vascular disease, cerebrovascular accident (CVA)

^b^ Structural heart disease was defined as either valvular heart disease or cardiomyopathy
