# Supplementary material for: COVID-19 associated hospitalization in 571 patients with fibromyalgia—A population-based study
Source: PLoS One. 2021 Dec 30;16(12):e0261772. doi: 10.1371/journal.pone.0261772 (PMC8717981; doi:10.1371/journal.pone.0261772)
Supplement: Attachment — Submitted filename: response to reviwers.docx [file pone.0261772.s002.docx]

## Saturday, October 23, 2021

## PLOS1 - Editor

Dear Editor,

Thank you for considering our manuscript “COVID-19 associated hospitalization and mortality in 571 patients with fibromyalgia - A population-based study” [PONE-D-21-20356].

We thank the reviewers for their efforts, we will present in this letter the modifications that had been done in our manuscript according to their comments.

\# reviewer 1

***I am confused about the outcome of this research. In the Method part, authors defined the “COVID-19 hospitalization” as the outcome, whereas from the title or conclusion, we can see “COVID-19 hospitalization and/or related mortality” was the outcome. Please clarify this point.***

We agree with the reviewer, since there was a small number of deceased, we clarified the outcome in the title and omitted the term mortality.

***As authors mentioned “We also formed a control group, electing for each FM patient two controls adjusted by age, gender and geographic location”, how did you make the match? Where did you choose the controls?***

We accept this comment and added the following statement “The controls were selected by a computerized algorithm that was applied on the database. The diagnosis of FM did not appear in their computerized medical records. The algorithm was provided selection of individuals that will have a similar distribution of their demographic parameters.” We added this clarification to the text (page 4).

***3.   Please check whether the variables “hospitalization duration” and “COVID-19 duration” follows normal distribution. If not, it is not proper to use mean (SD) to describe it.***
Thank you for the comment. These continuous variables do not follow normal distribution and their description was changed to median (IQR).

***4.   Authors conducted different multivariable logistic regressions in FM patients and controls, respectively. I think performing multivariable logistic regressions in the whole cohort (combining the FM patients and controls) would be preferable.***

The aim of this study was to investigate risk factors for poor COVID-19 related outcomes in patients with fibromyalgia and to investigate whether fibromyalgia for itself is a risk factor of that kind. For the second purpose mentioned we used a multivariable model in the whole cohort as suggested, we added a footnote to the Table 2 to further clarify that issue. For the first purpose such a model is irrelevant in our opinion as we did not intend to discover general risk factors for COVID-19 related poor outcomes (nor do the study population is representative for such purpose). Nonetheless, we added a table addressing the entire cohort in the supplementary materials (page 6).

\# reviewer 2

***In the introduction, it is not clear that the COVID-19 patients also experienced fibromyalgia.***

Thank you for the comment, we clarified this issue on page 3.

***Please explain why the fibromyalgia and control group were chosen as 1 to 2 rather than 1 to 1.***

Choosing multiple controls for a given number of cases increase the power of the study and enables to detect minor associations and in different subgroups.

***Amital at al please clarify how the control group was chosen since the patients also have similar conditions, e.g. rheumatoid arthritis, as you mentioned fibromyalgia was common rheumatology condition. What was the exclusion criteria for FM and control group?***

As mentioned above in our comment to reviewer 1 that we applied a computerized algorithm to all subjects in the same HMO that did not have fibromyalgia, their selection was based on obtaining similar distribution of demographic parameters. All these individuals never had the diagnosis of fibromyalgia included in their medical records (see page 4).

***What were the levels of the inflammatory biomarkers, e.g. IL-6, IL-8, CRP…?***

Unfortunately, these parameters were not available for our analysis. It should be mentioned that cytokine levels are not part of the routine tests in our country.

***Please define the abbreviates first, e.g. HCV and use accurate term “peripheral arterial disease” rather than “peripheral heart disease”.***

We thank the reviewer for the comment, these aspects were corrected in the text.

We thank the reviewers for their efforts, we modified the text accordingly and hope that paper will find in its new form appealing to the readership.

Sincerely yours

Howard Amital, MD, MHA
Head, Department of Medicine 'B'
Sheba Medical Center, (Affiliated to Tel-Aviv University)
Tel-Hashomer 5262100, Israel
 Tel: 972-3-5302652, Fax: 972-3-5304796

E-mail: Howard.amital@sheba.health.gov.il
