# Supplementary material for: COVID-19 associated hospitalization in 571 patients with fibromyalgia—A population-based study
Source: PLoS One. 2021 Dec 30;16(12):e0261772. doi: 10.1371/journal.pone.0261772 (PMC8717981; doi:10.1371/journal.pone.0261772)
Supplement: Peer review history

# Peer review: Decision Letter 0

**DOI:** 10.1371/journal.pone.0261772.r001

**Authors:** Ying-Mei Feng

8 Oct 2021

PONE-D-21-20356

COVID-19 associated hospitalization and mortality in 571 patients with fibromyalgia - A population-based study

PLOS ONE

Dear Dr. Amital,

Thank you for submitting your manuscript to PLOS ONE. After careful consideration, we feel that it has merit but does not fully meet PLOS ONE’s publication criteria as it currently stands. Therefore, we invite you to submit a revised version of the manuscript that addresses the points raised during the review process.

# reviewer 1

Mor Amital et al in their manuscript entitled "COVID-19 associated hospitalization and mortality in 571 patients with fibromyalgia - A population-based study" tried to explore where the presence of fibromyalgia (FM) are associated with

a severe COVID-19 disease course.

However, there are some major concerns.

1.   I am confused about the outcome of this research. In the Method part, authors defined the “COVID-19 hospitalization” as the outcome, whereas from the title or conclusion, we can see “COVID-19 hospitalization and/or related mortality” was the outcome. Please clarify this point.

2.   As authors mentioned “We also formed a control group, electing for each FM patient two controls adjusted by age, gender and geographic location”, how did you make the match? Where did you choose the controls?

3.   Please check whether the variables “hospitalization duration” and “COVID-19 duration” follows normal distribution. If not, it is not proper to use mean (SD) to describe it.

4.   Authors conducted different multivariable logistic regressions in FM patients and controls, respectively. I think performing multivariable logistic regressions in the whole cohort (combining the FM patients and controls ) would be preferable.

# reviewer 2

Amital et al in their manuscript entitled "COVID-19 associated hospitalization and mortality in 571 patients with fibromyalgia - A population-based study" tried to find the association of fibromyalgia and COVID-19 hospitalization or related mortality in the COVID-19 patients. It is an interesting study. However, there are some major concerns.

1.     In the introduction, it is not clear that the COVID-19 patients also experienced fibromyalgia.

2.     Please explain why the fibromyalgia and control group were chosen as 1 to 2 rather than 1 to 1.    

3.     Amital at al please clarify how the control group was chosen since the patients also have similar conditions, e.g. rheumatoid arthritis, as you mentioned fibromyalgia was common rheumatology condition. What was the exclusion criteria for FM and control group?

4.     What were the levels of the inflammatory biomarkers, e.g. IL-6, IL-8, CRP…?

5.     Please define the abbreviates first, e.g. HCV and use accurate term “peripheral arterial disease” rather than “peripheral heart disease”.

Please submit your revised manuscript by Nov 06 2021 11:59PM. If you will need more time than this to complete your revisions, please reply to this message or contact the journal office at plosone@plos.org. When you're ready to submit your revision, log on to https://www.editorialmanager.com/pone/ and select the 'Submissions Needing Revision' folder to locate your manuscript file.

Please include the following items when submitting your revised manuscript:

- A rebuttal letter that responds to each point raised by the academic editor and reviewer(s). You should upload this letter as a separate file labeled 'Response to Reviewers'.
- A marked-up copy of your manuscript that highlights changes made to the original version. You should upload this as a separate file labeled 'Revised Manuscript with Track Changes'.
- An unmarked version of your revised paper without tracked changes. You should upload this as a separate file labeled 'Manuscript'.

If you would like to make changes to your financial disclosure, please include your updated statement in your cover letter. Guidelines for resubmitting your figure files are available below the reviewer comments at the end of this letter.

If applicable, we recommend that you deposit your laboratory protocols in protocols.io to enhance the reproducibility of your results. Protocols.io assigns your protocol its own identifier (DOI) so that it can be cited independently in the future. For instructions see: https://journals.plos.org/plosone/s/submission-guidelines#loc-laboratory-protocols. Additionally, PLOS ONE offers an option for publishing peer-reviewed Lab Protocol articles, which describe protocols hosted on protocols.io. Read more information on sharing protocols at https://plos.org/protocols?utm_medium=editorial-email&utm_source=authorletters&utm_campaign=protocols.

We look forward to receiving your revised manuscript.

Kind regards,

Ying-Mei Feng

Academic Editor

PLOS ONE

Journal requirements:

When submitting your revision, we need you to address these additional requirements.

1. Please ensure that your manuscript meets PLOS ONE's style requirements, including those for file naming. The PLOS ONE style templates can be found at

https://journals.plos.org/plosone/s/file?id=wjVg/PLOSOne_formatting_sample_main_body.pdf and

https://journals.plos.org/plosone/s/file?id=ba62/PLOSOne_formatting_sample_title_authors_affiliations.pdf.

2. In the methods section of your manuscript, please thoroughly describe how the diagnosis of fibromyalgia was established in your cohort

3. Thank you for stating the following financial disclosure:

“this study was not sponsored by any party”

At this time, please address the following queries:

a) Please clarify the sources of funding (financial or material support) for your study. List the grants or organizations that supported your study, including funding received from your institution.

b) State what role the funders took in the study. If the funders had no role in your study, please state: “The funders had no role in study design, data collection and analysis, decision to publish, or preparation of the manuscript.”

c) If any authors received a salary from any of your funders, please state which authors and which funders.

d) If you did not receive any funding for this study, please state: “The authors received no specific funding for this work.

4. Thank you for stating the following in your Competing Interests section: 

“NO authors have competing interests”

Please complete your Competing Interests on the online submission form to state any Competing Interests. If you have no competing interests, please state ""The authors have declared that no competing interests exist."", as detailed online in our guide for authors at http://journals.plos.org/plosone/s/submit-now

 This information should be included in your cover letter; we will change the online submission form on your behalf.

Please include your amended statements within your cover letter; we will change the online submission form on your behalf.

5. We note that you have stated that you will provide repository information for your data at acceptance. Should your manuscript be accepted for publication, we will hold it until you provide the relevant accession numbers or DOIs necessary to access your data. If you wish to make changes to your Data Availability statement, please describe these changes in your cover letter and we will update your Data Availability statement to reflect the information you provide.

6. PLOS requires an ORCID iD for the corresponding author in Editorial Manager on papers submitted after December 6th, 2016. Please ensure that you have an ORCID iD and that it is validated in Editorial Manager. To do this, go to ‘Update my Information’ (in the upper left-hand corner of the main menu), and click on the Fetch/Validate link next to the ORCID field. This will take you to the ORCID site and allow you to create a new iD or authenticate a pre-existing iD in Editorial Manager. Please see the following video for instructions on linking an ORCID iD to your Editorial Manager account: https://www.youtube.com/watch?v=_xcclfuvtxQ

# Author comment: Author response to Decision Letter 0

**DOI:** 10.1371/journal.pone.0261772.r002

7 Dec 2021

Saturday, October 23, 2021

PLOS1 - Editor

Dear Editor, 

Thank you for considering our manuscript “COVID-19 associated hospitalization and mortality in 571 patients with fibromyalgia - A population-based study” [PONE-D-21-20356].

We thank the reviewers for their efforts, we will present in this letter the modifications that had been done in our manuscript according to their comments.

# reviewer 1

I am confused about the outcome of this research. In the Method part, authors defined the “COVID-19 hospitalization” as the outcome, whereas from the title or conclusion, we can see “COVID-19 hospitalization and/or related mortality” was the outcome. Please clarify this point.

We agree with the reviewer, since there was a small number of deceased, we clarified the outcome in the title and omitted the term mortality. 

As authors mentioned “We also formed a control group, electing for each FM patient two controls adjusted by age, gender and geographic location”, how did you make the match? Where did you choose the controls?

We accept this comment and added the following statement “The controls were selected by a computerized algorithm that was applied on the database. The diagnosis of FM did not appear in their computerized medical records. The algorithm was provided selection of individuals that will have a similar distribution of their demographic parameters.” We added this clarification to the text (page 4). 

3. Please check whether the variables “hospitalization duration” and “COVID-19 duration” follows normal distribution. If not, it is not proper to use mean (SD) to describe it.

Thank you for the comment. These continuous variables do not follow normal distribution and their description was changed to median (IQR).

4. Authors conducted different multivariable logistic regressions in FM patients and controls, respectively. I think performing multivariable logistic regressions in the whole cohort (combining the FM patients and controls) would be preferable.

The aim of this study was to investigate risk factors for poor COVID-19 related outcomes in patients with fibromyalgia and to investigate whether fibromyalgia for itself is a risk factor of that kind. For the second purpose mentioned we used a multivariable model in the whole cohort as suggested, we added a footnote to the Table 2 to further clarify that issue. For the first purpose such a model is irrelevant in our opinion as we did not intend to discover general risk factors for COVID-19 related poor outcomes (nor do the study population is representative for such purpose). Nonetheless, we added a table addressing the entire cohort in the supplementary materials (page 6). 

# reviewer 2

In the introduction, it is not clear that the COVID-19 patients also experienced fibromyalgia.

Thank you for the comment, we clarified this issue on page 3. 

Please explain why the fibromyalgia and control group were chosen as 1 to 2 rather than 1 to 1. 

Choosing multiple controls for a given number of cases increase the power of the study and enables to detect minor associations and in different subgroups.

Amital at al please clarify how the control group was chosen since the patients also have similar conditions, e.g. rheumatoid arthritis, as you mentioned fibromyalgia was common rheumatology condition. What was the exclusion criteria for FM and control group?

As mentioned above in our comment to reviewer 1 that we applied a computerized algorithm to all subjects in the same HMO that did not have fibromyalgia, their selection was based on obtaining similar distribution of demographic parameters. All these individuals never had the diagnosis of fibromyalgia included in their medical records (see page 4).

What were the levels of the inflammatory biomarkers, e.g. IL-6, IL-8, CRP…?

Unfortunately, these parameters were not available for our analysis. It should be mentioned that cytokine levels are not part of the routine tests in our country. 

Please define the abbreviates first, e.g. HCV and use accurate term “peripheral arterial disease” rather than “peripheral heart disease”.

We thank the reviewer for the comment, these aspects were corrected in the text. 

We thank the reviewers for their efforts, we modified the text accordingly and hope that paper will find in its new form appealing to the readership. 

Sincerely yours

Howard Amital, MD, MHA

Attachment Submitted filename: response to reviwers.docx Click here for additional data file.

# Editor report: Decision Letter 1

**DOI:** 10.1371/journal.pone.0261772.r003

**Authors:** Ying-Mei Feng

10 Dec 2021

COVID-19 associated hospitalization in 571 patients with fibromyalgia - A population-based study

PONE-D-21-20356R1

Dear Dr. Amital,

We’re pleased to inform you that your manuscript has been judged scientifically suitable for publication and will be formally accepted for publication once it meets all outstanding technical requirements.

Within one week, you’ll receive an e-mail detailing the required amendments. When these have been addressed, you’ll receive a formal acceptance letter and your manuscript will be scheduled for publication.

An invoice for payment will follow shortly after the formal acceptance. To ensure an efficient process, please log into Editorial Manager at http://www.editorialmanager.com/pone/, click the 'Update My Information' link at the top of the page, and double check that your user information is up-to-date. If you have any billing related questions, please contact our Author Billing department directly at authorbilling@plos.org.

If your institution or institutions have a press office, please notify them about your upcoming paper to help maximize its impact. If they’ll be preparing press materials, please inform our press team as soon as possible -- no later than 48 hours after receiving the formal acceptance. Your manuscript will remain under strict press embargo until 2 pm Eastern Time on the date of publication. For more information, please contact onepress@plos.org.

Kind regards,

Ying-Mei Feng

Academic Editor

PLOS ONE

# Editor report: Acceptance letter

**DOI:** 10.1371/journal.pone.0261772.r004

**Authors:** Ying-Mei Feng

17 Dec 2021

PONE-D-21-20356R1 

COVID-19 associated hospitalization in 571 patients with fibromyalgia - A population-based study 

Dear Dr. Amital:

I'm pleased to inform you that your manuscript has been deemed suitable for publication in PLOS ONE. Congratulations! Your manuscript is now with our production department. 

If your institution or institutions have a press office, please let them know about your upcoming paper now to help maximize its impact. If they'll be preparing press materials, please inform our press team within the next 48 hours. Your manuscript will remain under strict press embargo until 2 pm Eastern Time on the date of publication. For more information please contact onepress@plos.org.

If we can help with anything else, please email us at plosone@plos.org. 

Thank you for submitting your work to PLOS ONE and supporting open access. 

Kind regards, 

PLOS ONE Editorial Office Staff

on behalf of

Dr Ying-Mei Feng 

Academic Editor

PLOS ONE
